# Protocol of the Temporality, Eating and Metabolic health during PreconceptiOn (TEMPO) study in females with overweight and obesity: a prospective observational cohort nested within a preconception lifestyle intervention program

**DOI:** 10.1186/s12978-026-02347-4

**Published:** 2026-04-30

**Authors:** See Ling Loy, Chee Wai Ku, Jun Wei Pek, Yin Bun Cheung, Melvin Khee Shing Leow, Tuck Seng Cheng, Mary Foong-Fong Chong, Keith M Godfrey, Fabian Yap, Jerry Kok Yen Chan

**Affiliations:** 1 Department of Reproductive Medicine, KK Women’s and Children’s Hospital, Singapore, 229899 Singapore; 2 Academic Clinical Program, Duke-NUS Medical School, Singapore, Singapore; 3 SingHealth Duke-NUS Maternal and Child Health Research Institute, KK Women’s and Children’s Hospital, Singapore, Singapore; 4 Temasek Life Sciences Laboratory, Singapore, Singapore; 5 Department of Biological Sciences, National University of Singapore, Singapore, Singapore; 6 Program in Health Services & Systems Research and Centre for Quantitative Medicine, Duke-NUS Medical School, Singapore, Singapore; 7 Tampere Centre for Child, Adolescent and Maternal Health Research, Tampere University, Tampere, Finland; 8 Endocrinology, Tan Tock Seng Hospital, Singapore, Singapore; 9 Institute for Human Development and Potential, Singapore Agency for Science, Technology and Research (A*STAR), Singapore, Singapore; 10 Lee Kong Chian School of Medicine, Nanyang Technological University, Singapore, Singapore; 11 National Perinatal Epidemiology Unit, Nuffield Department of Population Health, University of Oxford, Oxford, United Kingdom; 12 Saw Swee Hock School of Public Health, National University of Singapore, Singapore, Singapore; 13 Medical Research Council Lifecourse Epidemiology Centre, University of Southampton, Southampton, United Kingdom; 14 National Institute for Health Research Southampton Biomedical Research Centre, University of Southampton and University Hospital Southampton National Health Service Foundation Trust, Southampton, United Kingdom; 15 Endocrinology Service, KK Women’s and Children’s Hospital, Singapore, Singapore

**Keywords:** Behavior, Chronobiology, Chrononutrition, Clock gene, Female reproductive health, Meal timing, Preconception, Sleep

## Abstract

**Introduction:**

Human reproduction is tightly regulated by circadian and metabolic signals. However, the extent to which alterations in these systems affect fertility, especially in females with overweight or obesity who are at increased risk of infertility, remains poorly understood. This study, nested within the Healthy Early Life Moments in Singapore (HELMS) integrated lifestyle intervention program, aims to: (i) examine the associations between changes in circadian behavior and metabolic health indicators over a 3-month period and subsequent reproductive outcomes during a year of conception attempts, (ii) identify risk factors associated with these changes, and (iii) elucidate the biological mechanisms underpinning these relationships in females attempting to conceive.

**Methods:**

This prospective observational cohort study will enroll 283 females with a BMI of 25–40 kg/m² participating in the HELMS program at KK Women’s and Children’s Hospital, Singapore. Participants will be followed for one year as they attempt to conceive. At baseline and at the 3-month mark, circadian behavior will be assessed using validated questionnaires, tracking diaries, and digital wearables. Metabolic health will be evaluated through anthropometry, body fat composition, a metabolic syndrome score, and an insulin resistance index. Blood samples will be collected to analyze metabolic biomarkers and gene expression levels. We will use modified Poisson regression models to examine associations with the clinical pregnancy rate (primary outcome) and discrete-time proportional hazards models to estimate associations with fecundability (secondary outcome) within one year of conception attempts.

**Discussion:**

This study is pivotal for identifying potential novel modifiable risk factors to address low fertility rates. Insights from this research will generate hypotheses for interventions designed to enhance preconception care. By screening and managing circadian behaviors and metabolic profiles among females with overweight and obesity, these strategies may benefit those experiencing unexplained infertility. Ultimately, this approach could foster a shift towards a more holistic and patient-centered model of reproductive healthcare.

## Plain language summary

Human reproduction is shaped by the body’s internal clock and metabolism, but it is not yet clear how changes in these systems affect fertility, particularly in women with overweight or obesity, who are at higher risk of infertility. This study, part of the Healthy Early Life Moments in Singapore (HELMS) program, aims to better understand these links. A total of 283 women with a body mass index between 25 and 40 kg/m^2^ will be recruited at KK Women’s and Children’s Hospital, Singapore, and followed for one year while trying to conceive. At baseline and 3-month follow-up, participants’ daily routines, including the timing of eating and sleep, will be recorded using questionnaires, diaries, and wearable devices. Their metabolic health will be assessed through body fat, waist size, blood sugar control, and blood tests for biomarkers and gene activity. Researchers will analyze how changes in these patterns relate to the chances of becoming pregnant and the time it takes to conceive. This work is important because it may reveal lifestyle and health factors that can be modified to improve fertility. The findings will help shape new approaches to support women before pregnancy. By addressing daily routines such as meal timing and sleep, alongside metabolic health, this study may offer practical strategies for women struggling to conceive without a clear medical cause identified through standard reproductive investigations. In the long run, this approach could lead to more personalized and supportive fertility care that looks beyond medical treatment to the whole person.

## Background

Singapore has grappled with a persistently low total fertility rate of 1.1 since 2018, which dropped to 0.97 in 2024 [1]. Despite comprehensive pronatalist policies, this rate remains one of the lowest globally, akin to many developed countries in the region [2]. This situation is complicated by biological factors, notably the sensitivity of the hypothalamic-pituitary-gonadal axis, which tightly regulates human reproduction, to circadian and metabolic cues [3]. While social factors are influential, the persistently low fertility rates in Singapore may also be partly due to increasing disrupted circadian rhythms and escalating obesity rates, stemming from unhealthy and erratic lifestyles among the population [4, 5]. This notion is supported by reviews consistently demonstrating lower pregnancy rates among shift workers and delayed pregnancies in females with excessive weight [6, 7]. Notably, overweight and obesity are increasingly common at preconception and are associated with reduced fecundability even in the absence of diagnosed infertility [8].

Besides shift work, other environmental disruptors of circadian rhythms, such as improper meal timing, sleep deprivation, and irregular sleep-wake patterns, have been associated with reduced fertility, potentially through suppression of circadian clock gene expression [3]. However, beyond sleep disturbances, the effects of unhealthy practices related to chrononutrition and chronobiology, including metabolic jet lag (a discrepancy in eating times between workdays and rest days), social jet lag (a discrepancy in sleep times between workdays and rest days), extended eating windows, and late-night eating on fertility, have not been thoroughly investigated, despite their prevalence in modern societies [9, 10]. Whether these behaviors translate into measurable metabolic perturbations relevant to reproductive outcomes in the preconception period remains unclear, and human preconception data integrating time-resolved circadian behaviors, metabolic biomarkers, and subsequent reproductive outcomes are limited.

A recent meta-analysis of randomized controlled trials revealed that while successful weight loss increased the chances of pregnancy in females with overweight or obesity, the highest weight loss did not necessarily correspond with the highest pregnancy and live birth rates [11]. Additionally, our prior study reported that females with high body mass index (BMI ≥ 23 kg/m^2^) who exhibited low insulin resistance or absence of metabolic syndrome had shorter time-to-pregnancy intervals compared with their metabolically unhealthier counterparts [12]. These findings indicate that females with overweight or obesity are a metabolically heterogeneous group, and that reproductive potential may be more closely linked to metabolic health status than to body weight per se. While animal models have provided supportive evidence for the role of metabolic hormones in correcting sterility in homozygous obese female mice [13], a lack of corresponding human data hampers the development of targeted fertility-enhancement strategies. Currently, clinical approaches predominantly focus on managing weight and BMI [11].

**Fig. 1:**
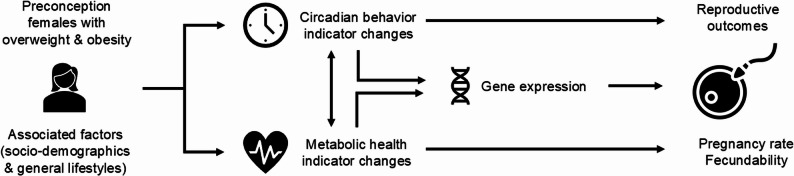
Study conceptual framework. The diagram illustrates the relationships between circadian behavior and metabolic health indicator changes, and subsequent reproductive outcomes, specifically pregnancy rate and fecundability, among preconception females with overweight and obesity. It also investigates associated risk factors based on socio-demographics and general lifestyles, and delves into the underlying molecular mechanisms through gene expression analysis

Taken together, the impact of circadian and metabolic disruptions prior to conception on female reproductive outcomes, especially among those with overweight or obesity who face a higher risk of infertility, remains poorly understood. The mechanisms and extent of these effects on fertility have not been adequately explored, constraining the development and implementation of effective interventions and educational programs in both public and clinical settings. This gap underscores a significant unmet need in preconception care and forms the primary motivation for this study. Therefore, our goals are to identify specific modifiable circadian behavior and metabolic health indicators that potentially contribute to reproductive outcomes, which can be targeted for intervention to improve reproductive health in preconception females. By additionally exploring changes in circadian- and metabolism-related gene expression, this study seeks to provide biological context linking circadian behavioral and metabolic changes to reproductive outcomes. Figure 1 illustrates the conceptual framework of the present study.

### Objectives

The overall objective of this study is to determine how changes in circadian behavior and metabolic health indicators are associated with reproductive health outcomes in females with overweight or obesity who are attempting to conceive within the HELMS program. Additionally, this study seeks to identify associated risk factors and explore the biological mechanisms underlying these relationships. We hypothesize that desirable changes in circadian behavior and metabolic health indicators are associated with favorable reproductive outcomes in preconception females with overweight and obesity. These changes are expected to correlate with specific characteristics of the females and are accompanied by concurrent alterations in the expression levels of specific genes, particularly those related to circadian rhythms, metabolism, and fertility. The specific objectives are:

1. To examine the association between changes in circadian behavior (meal and sleep timing) and metabolic health indicators (BMI, body fat composition, and metabolic biomarkers) over a 3-month period, and subsequent reproductive outcomes (clinical pregnancy rate and fecundability) within one-year of conception attempts among preconception females with overweight or obesity.
2. To identify baseline socio-demographic and lifestyle factors associated with changes in circadian behavior and metabolic health indicators among these females.
3. To explore the relationship between concurrent changes in circadian behavior and metabolic health indicators, and gene expression levels.

## Methods/design

### Study design and setting

We conduct a prospective observational preconception cohort study, named Temporality, Eating and Metabolic health during PreconceptiOn (TEMPO), among females with overweight or obesity who are attempting to conceive over a 12-month period from recruitment. TEMPO is nested within the ongoing Healthy Early Life Moments in Singapore (HELMS) integrated lifestyle intervention program, which was initiated in March 2022 at KK Women’s and Children’s Hospital, Singapore [14]. TEMPO was introduced into HELMS in March 2024.

### Participants and recruitment

Participants who meet eligibility criteria are recruited from the HELMS starting from the baseline visit, with a one-year follow-up period. Recruitment is expected to be completed by December 2025, and follow-up data collection by January 2027.

### Inclusion criteria

1. Females aged 21–40 years.
2. BMI between 25 and 40 kg/m^2^ (those with BMI > 40 kg/m^2^ are excluded due to special clinical needs and potential bariatric surgery considerations).
3. Of Chinese, Malay, Indian ethnicity, or any combination thereof.
4. Planning to conceive within one year.
5. Ability to understand English.
6. Access a mobile app on iOS or Android platform.
7. Provision of written, informed consent.

### Exclusion criteria

1. Currently pregnant.
2. Known type 1 or type 2 diabetes.
3. Use of any anticonvulsant medication within the past month.
4. Use of any oral steroid within the past month.
5. Use of any oral, injectable, patch or implanted contraception or an intrauterine contraceptive device in situ within the past month.
6. Use of any fertility medication other than Clomid/ Letrozole (Femara) within the past month.
7. Use of HIV or Hepatitis B or C medications within the past month.
8. History of gynecological masses or tumors.

### Patient and public involvement

The development of the current TEMPO study protocol was informed by feedback from participants in the ongoing HELMS study. This feedback process allowed us to ascertain participant preferences that significantly influenced the refinement of our study workflow. Specifically, participants’ willingness to respond additional questionnaires, attend an additional 3-month physical visit, undergo further blood collections, and their preferences for visit timings have critically shaped both the design and assessment strategies of this study.

### HELMS integrated lifestyle intervention

HELMS is conceptualized as a new model-of-care that employs an early life course approach through a mobile health platform to transform maternal and child health in Singapore [15]. The HELMS study is designed as an implementation trial to assess the efficacy of this innovative model in optimizing the metabolic and mental health of females with overweight or obesity throughout the preconception, pregnancy, and postpartum periods. Details of the HELMS protocol have been published elsewhere [14]. In brief, the intervention components specific to the preconception phase, which are similarly experienced by all TEMPO participants, include the following: After initial baseline assessments, each participant consults a doctor who provides in-person consultations and prescribes micronutrient supplements. The HELMS mobile app is introduced to provide educational materials on self-care, sexual health, and healthy lifestyles. Equipped with feedback and goal-setting functions, the app utilizes participants’ data inputs to generate reports and recommendations, guiding them towards a healthier behavior. Weekly motivational nudges are sent via the app throughout one year of conception attempts to offer anticipatory guidance in preconception care and encourage healthy lifestyle habits.

### Study procedure

**Fig. 2:**
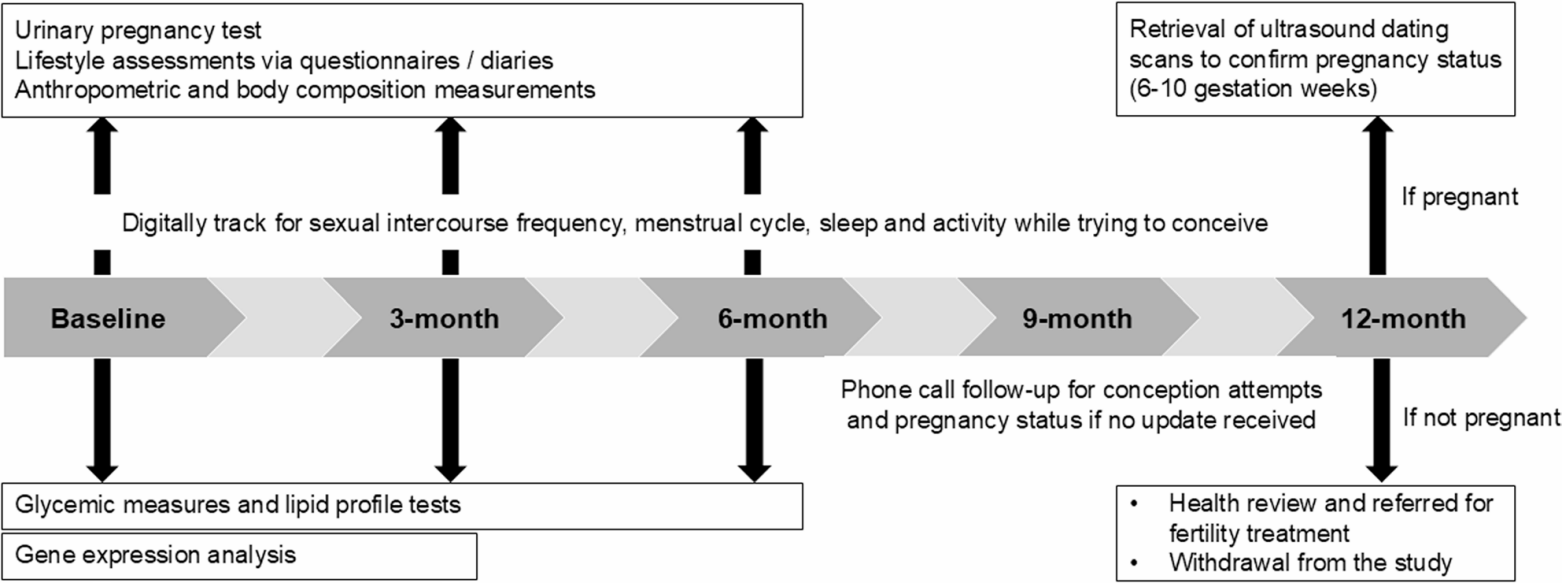
Study workflow and measurements. Baseline, 3-month, and 6-month visits include the administration of questionnaires or diaries, physical measurements, and blood tests. Participants track their frequency of sexual intercourse, menstrual periods, sleep, and activity using a mobile application and digital wearable while trying to conceive. The 9-month visit consists of a phone call where research staff check participants’ pregnancy status and conception attempts if no update received. Throughout the follow-up period, participants with a positive urinary pregnancy test are invited to undergo a dating scan at 6–10 gestation weeks to confirm pregnancies, with outcomes retrieved from medical records. Participants who do not conceive by 12-month will be invited to attend a medical review and referred for fertility medical treatment before withdrawal from the study

Figure 2 illustrates the study workflow. The study begins with participants providing written informed consent at the HELMS baseline visit. During this visit, participants complete questionnaires assessing socio-demographics, medical history, and lifestyle factors. Socio-demographic data include age, ethnicity, education, household income, and employment status. Medical histories focus on chronic diseases and obstetric history, while lifestyle factors cover alcohol intake, smoking status, physical activity (International Physical Activity Questionnaire) [16], nutritional behaviors (Food Frequency Questionnaire or Diet Screener, and 6P Nutrition Tool) [17–19], emotional health (Edinburgh Postnatal Depression Scale) [20], and sleep quality (Pittsburgh Sleep Quality Index) [21]. Clinical measurements such as blood pressure, height, weight, body fat composition, and waist circumference are taken using standardized equipment and are recorded in duplicate. Participants undergo a standard 75 g oral glucose tolerance test and provide blood samples, which will be analyzed for glucose, insulin, and lipid profiles within two hours of collection. Additional blood samples are collected for gene expression analysis and storage for future analyses.

At the same visit, each participant receives a digital wearable ‘Oura Ring’ (Oura Health Oy, Finland) for continuous monitoring of sleep and activity. The participants are guided on how to use the HELMS mobile app to track dietary intake, sleep-wake patterns, sexual activity, and menstrual cycles, with a paper diary available as an alternative. Participants are instructed to promptly report any positive pregnancy tests after the baseline visit to schedule an ultrasound confirmation. Physical follow-up visits, which include the same assessments as at baseline, are scheduled at 3-month and 6-month intervals if conception has not occurred, during which conception attempts and family planning decisions are monitored. At 9-month, research staff conduct a follow-up phone call to check on conception attempts and pregnancy status. By 12-month, non-pregnant participants undergo a final health review and are referred for fertility treatment if needed.

Partner assessments are conducted once at the baseline visit during the preconception period to collect socio-demographics, medical history, chrononutrition profile, weight, height, and body fat composition; semen analysis results are retrieved from medical records, if available. Detailed data collection is summarized in Table 1.

**Table 1:** Data collection

	Baseline	3-month	6-month	9-month	12-month
Informed consent	√				
Eligibility criteria	√				
Background characteristics^a^					
Socio-demographics	√				
Medical history	√				
Lifestyles					
Alcohol intake and cigarette smoking	√	√ ^b^	√		
Physical activity	√	√ ^b^	√		
Dietary pattern	√	√ ^b^	√		
Emotional health	√	√ ^b^	√		
Sleep quality	√	√ ^b^	√		
Circadian behavior					
Chrononutrition profile^a^	√	√ ^b^	√ ^b^		
Chronotype	√				
Food and sleep diary	√				
Digital wearable (sleep & activity)	√ ^b^ (throughout the period of trying to conceive)				
Metabolic health					
Blood pressure	√	√ ^b^	√		
Height*	√	√ ^b^	√		
Weight and body composition^a^	√	√ ^b^	√		
Fasting plasma glucose	√	√ ^b^	√		
2-hour plasma glucose	√				
Fasting serum insulin	√	√ ^b^	√		
Fasting serum lipid panel	√	√ ^b^	√		
Reproductive health					
Serum Anti-Müllerian Hormone	√				
Pregnancy status and attempts	√	√	√	√	√
Menstrual cycle & sex frequency	√	√	√	√	√
Ultrasound dating scan (if pregnant)	√ (6–10 gestation weeks)				
Gene expression analysis	√ ^b^	√ ^b^			

^a^Measurements which are similarly performed on male partners

^b^The additional measurements introduced in the TEMPO study, which complement the existing measurements in HELMS

### Circadian behavior assessment

Circadian behavior is assessed using the Chrononutrition Profile Questionnaire [22, 23], which evaluates key variables such as metabolic jet lag, social jet lag, nocturnal eating, breakfast skipping, eating windows, and sleep duration. The Chrononutrition Profile Questionnaire, comprising 18 items, captures general patterns of chrononutrition preferences and behaviors on typical work/school days and free days. It includes questions on meal timing, frequency of meals and snacks, sleep-wake times, and the type of the largest meal intake. Desirable changes in circadian behavior are defined as reductions in metabolic and social jet lag, nocturnal eating, breakfast skipping, and eating window duration, together with shifts towards longer sleep duration, as well as earlier meal and sleep timing. Additionally, chronotype will be determined using the Morningness-Eveningness Questionnaire consists of 19 items, each rated on a 4 or 5-point numerical scale, with total scores ranging from 16 to 86; higher scores indicate a morning-type tendency [24]. The accuracy of these self-reported measures will be enhanced by cross verifying the same variables using food and sleep diaries (covering three workdays and one rest day) and data from digital wearables.

### Metabolic health assessment

BMI is calculated by dividing an individual’s weight in kilograms by the square of their height in meters. Body fat percentage and distribution are measured using the InBody 970 Body Composition Analyzer (InBody, Korea), which employs bioelectrical impedance analysis technology and direct segmental measurement. A metabolic score is derived based on metabolic syndrome criteria, awarding one point for each of the following conditions: (i) waist circumference ≥ 80 cm; (ii) triglyceride ≥ 1.7 mmol/L; (iii) high density lipoprotein-cholesterol < 1.3 mmol/L; (iv) fasting glucose ≥ 5.6 mmol/L; and (v) blood pressure ≥ 130/85 mmHg, or on treatment [25]. Insulin resistance is calculated based on the updated homeostasis model assessment of insulin resistance (HOMA2-IR) index, accessible via the HOMA2 calculator online at http://www.dtu.ox.ac.uk/homacalculator/ [26]. Desirable changes in metabolic health are defined as reductions in BMI, body fat percentage, visceral fat area, metabolic syndrome score, and insulin resistance.

### Gene expression analysis

Peripheral blood samples for gene expression analysis are collected in Tempus blood ribonucleic acid (RNA) tubes, from which total RNA will be extracted. RNA sequencing will be conducted on a selected subset of samples to identify candidate genes. Subsequently, reverse transcription-quantitative real-time polymerase chain reaction will be performed on all other samples to validate these findings. To quantify changes in gene expression, the 2 − ΔΔCt method will be used, comparing gene expression levels at baseline with those at the 3-month follow-up.

### Outcomes and assessments

The primary outcome of the study is the clinical pregnancy rate, defined as the probability of successful conception within 12 months from enrolment, whether spontaneous or through assisted reproductive technology. A clinical pregnancy is confirmed by a positive urinary pregnancy test followed by the detection of an intrauterine gestational sac after six weeks of amenorrhea via an ultrasound scan [12]. If an ultrasound scan is not available or inconclusive, the diagnosis of pregnancy will be made clinically. Biochemical pregnancy is not counted as a pregnancy event [27].

The secondary outcome, fecundability, is defined as the probability of conceiving during a normal menstrual cycle with unprotected intercourse [28]. It is measured by the time-to-pregnancy in menstrual cycles, consistent with methodologies described in our previous studies [8, 12, 27]. Specifically, we calculate the interval between the date of the last menstrual period at recruitment (baseline) and before conception (for those who became pregnant within one year) or the last follow-up call (for those who did not conceive or withdrew from the study). This interval is then converted into menstrual cycles by dividing by the average cycle length. The number of months attempting to conceive prior to study entry is considered in the computation of time-to-pregnancy. Monthly menstrual and coital histories captured over the study period are used to determine the cycle length and the cycle-at-risk, minimizing the risk of overestimating the number of cycles at risk in the absence of sexual intercourse.

In addition, live birth rates will be tracked as a downstream reproductive outcome through linkage with medical records among participants who achieve clinical pregnancy.

### Statistical analysis plan

We will perform statistical analyses using the Stata Statistical Software. The primary exposure of interest in this study is the change in circadian behavior and metabolic health indicators over the first 3 months of the preconception period. Although the HELMS program continues throughout preconception, pregnancy, and the postnatal period, this initial 3-month interval is pre-specified in TEMPO as the primary exposure window. This interval was selected to allow sufficient time to capture measurable changes in circadian behaviors and metabolic indicators before pregnancy-related changes occur and before substantial loss to follow-up. Accordingly, only participants who complete the 3-month assessment have a fully characterized exposure and are included in the primary analysis, ensuring correct temporal ordering between exposure assessment and subsequent reproductive outcomes.

To investigate changes in circadian behavior and metabolic health indicators between baseline and the 3-month follow-up as exposure variables, the primary analysis will include only participants who have completed the 3-month visit. To identify any potential selection bias, we will compare baseline characteristics between participants included in and excluded from the primary analysis, as well as those recruited into the TEMPO and HELMS studies alone. We will use Pearson’s chi-squared test for categorical variables and either independent t-test or Mann-Whitney U test for continuous variables.

We will examine the associations of changes in circadian behavior and metabolic health indicators with pregnancy rates using modified Poisson regression models. These models will estimate risk ratios and 95% confidence intervals, adjusting for potential covariates identified from the literature and using a directed acyclic graph [6, 12, 29, 30]. Changes in these indicators will be standardized to z-scores to facilitate comparison of the relative impacts of each exposure indicator. These indicators will be treated either as continuous or categorical exposures (e.g. by tertiles or quantiles). Sensitivity analyses will re-evaluate models with spontaneous conception and live birth as the outcomes.

To examine the associations of circadian behavior and metabolic health indicator changes with fecundability, we will use discrete-time proportional hazards models. These models analyze time-to-pregnancy on a discrete scale (number of menstrual cycles-at-risk during active pregnancy attempts), to estimate the hazard ratio of fecundability, termed as the fecundability ratio, along with a 95% confidence interval [31]. Adjustments for potential covariates will be made, similar to those used in the above Poisson regression models [6, 12, 29, 30]. Unlike the hazard ratio for mortality or morbidity where a ratio of < 1 is desirable, a fecundability ratio of < 1 indicates an undesirable outcome which suggests delayed pregnancy. We will account for left truncation by basing our analysis on risk sets for observed cycles at risk, considering only the cycles actively attempted during the study period. For instance, if a participant has been trying to conceive for four cycles at study entry and achieves pregnancy after seven cycles, only the cycles from the fifth to the seventh will be included in the analysis. Participants will be censored in the analysis if they do not conceive after 12 months from recruitment, start fertility treatment, cease trying to conceive, or are lost to follow-up.

To analyze the associations of socio-demographic and lifestyle factors with circadian behavior and metabolic health changes, we will employ multivariable linear regression for continuous outcomes and multinomial logistic regression for categorical outcomes, adjusting for potential covariates. For the assessment of changes in circadian behavior and metabolic health indicators with gene expression levels, we will utilize independent t-tests or Mann-Whitney U test to evaluate differences in gene expression between groups categorized by high versus low health indicators, determined by median thresholds at each time point. Additionally, correlation testing (Pearson or Spearman) will identify associations between these variables and gene expression. Corrections for multiple testing, such as Bonferroni or false discovery rate, will be applied to reduce the Type I error rate.

A secondary, exploratory analysis will include only participants who have not conceived by the 6-month visit to examine long-term trajectories of circadian behavior and metabolic health indicators. We will use group-based trajectory modelling to characterize these changes at baseline, 3-month, and 6-month intervals, anticipating different patterns such as later improvement, worsening, or stability over the period. Each trajectory group will be analyzed for its association with pregnancy rates and fecundability, adjusting for potential covariates.

Missing data will be handled through multiple imputation, creation of a distinct category for the variable, or exclusion of the variable if the missingness exceeds a predefined threshold (> 15%). We will further perform sensitivity analysis by including only complete data in the analysis.

### Sample size

To address the primary aim based on the assumption to detect a medium effect size of 0.4 standard deviation (equivalent to approximately a one-unit change in BMI) in changes of circadian behavior or metabolic health indicators from baseline to 3-month, we calculated that a sample size of 198 females is required. This sample size is supported by findings from previous trials that employed the healthy plate concept or a digital intervention alone, both components of the HELMS intervention, which demonstrated significant reductions in BMI exceeding one unit over a 3-month period [32, 33]. The calculation was based on achieving 80% statistical power and maintaining a two-sided type I error rate of 5%, under the assumption that 50% of the cohort will become pregnant within the year. To account for a potential 30% incomplete 3-month follow-up (including an estimated 15% pregnancy rate within 3 months) and parameter uncertainty, we will enroll a total of 283 participants.

### Data management

To ensure data quality and minimize missingness, the study will employ a three-tiered data checking procedure. The first tier, using algorithms in the RedCap platform, detects outliers and missing data, serving as an early alert system for the clinical research staff to immediately address potential errors during participant visits. The second tier involves monthly monitoring of home diary records through Excel tracking logs. Any identified missing or suspicious data will be addressed during reminders or follow-up calls with participants. The third tier includes quarterly or ad-hoc data cleaning using statistical approaches, incorporating verification against medical records, clinical justifications, and team discussions for quality assurance.

### Ethics and dissemination

The Centralized Institutional Review Board of SingHealth approved both the initial HELMS protocol and additional data collection for this TEMPO study (reference number 2021/2247). Researchers will conduct the study in line with the ethical principles of the Declaration of Helsinki. All participants will provide written informed consent. The team plans to disseminate the study’s findings through peer-reviewed publications, conference presentations, and reports to the funding body. Data that support the findings of this study will be available from the corresponding author on reasonable request. After completing the study, the principal investigator will share the deidentified data with National Medical Research Council, Singapore, in accordance with the Research Data Governance and Sharing Framework.

## Discussion

The TEMPO is a prospective observational cohort study nested within the HELMS program in females with overweight or obesity who are attempting to conceive. This study aims to examine the associations between changes in circadian behavior and metabolic health indicators over a 3-month period and subsequent reproductive outcomes, while also understanding the associated factors and biological mechanisms underpinning these relationships. Comprehensive data are collected, integrating subjective self-reported questionnaires, objective measures from digital wearables and tracking diaries, and biomarkers including clinical blood tests and gene expression analysis. This multifaceted data collection approach is structured to provide robust observational evidence that may suggest avenues for optimizing preconception care and enhancing reproductive success among this population. As this study is not designed to include an external control cohort, observed conception rates will be interpreted in the context of previously published preconception fertility estimates from Singapore and comparable populations, including data from the Singapore PREconception Study of long-Term maternal and child Outcomes [34].

The insights gained from the TEMPO study could lead to hypotheses for interventions aimed at improving preconception care by screening and managing circadian behaviors and metabolic profiles among targeted females. Such strategies may be particularly beneficial for females facing unexplained infertility and could promote a shift toward more holistic and patient-centered reproductive healthcare. The extensive information connecting circadian behavior, metabolic health, and fertility from this study may expand the potential applications of chronotherapy targeting the circadian system and pharmacotherapy targeting metabolic hormones to improve reproductive health. Although the study focuses on females with overweight or obesity, the outcomes of this study could benefit others trying or struggling to conceive, as disturbances in circadian and metabolic regulations can occur at any individual, regardless of weight status [35].

Several limitations exist within this study. Due to its observational nature, the study may not definitively establish causal relationships between circadian and metabolic changes and reproductive outcomes. The reliance on self-reported data for certain lifestyle factors and circadian behaviors may introduce reporting biases. We are mitigating these biases by integrating data from digital wearables and diary records to validate and strengthen self-reported information. This multifaceted approach will not only confirm findings but also facilitate the practical application of screening tools derived from questionnaires for broader use in future. The loss to follow-up may introduce selection biases. We are minimizing dropout rates through several strategies, including maintaining good communication and rapport with participants, providing incentives for time reimbursement and continued participation, and offering home visit options if necessary. Additionally, while recruitment is limited to Singapore, the ethnically diverse sample may offer insights applicable to other Asian populations, though broader generalizability will require further validation.

## Data Availability

No datasets were generated or analysed during the current study.

## Abbreviations

BMI: Body mass index
HELMS: Healthy Early Life Moments in Singapore
HOMA2-IR: Updated homeostasis model assessment of insulin resistance
RNA: Ribonucleic acid
TEMPO: Temporality, Eating and Metabolic health during PreconceptiOn
